# The effects of physical inactivity on other risk factors for chronic disease: A systematic review of reviews

**DOI:** 10.1016/j.pmedr.2024.102866

**Published:** 2024-08-22

**Authors:** Emily Bourke, Jonathan Rawstorn, Ralph Maddison, Tony Blakely

**Affiliations:** a Population Interventions, Centre for Epidemiology and Biostatistics, Melbourne School of Population and Global Health, University of Melbourne, Melbourne, Australia; b Institute for Physical Activity and Nutrition, School of Exercise & Nutrition Sciences, Deakin University, Geelong, Australia

**Keywords:** Risk factors, Epidemiology, Physical activity, Cholesterol, Hypertension, Diabetes

## Abstract

**Background:**

The Global Burden of Disease (GBD) 2021 study updated methods for attributing burden to physical inactivity, to include all conditions from fasting plasma glucose (FPG) due to physical inactivity. However, physical inactivity influences several additional GBD risk factors that also effect other diseases. This study estimated effects of physical activity on high blood pressure (hypertension), FPG (as diabetes), osteoporosis, and LDL-cholesterol, to enable mediation effects modelling.

**Methods:**

MEDLINE, ProQuest Central, Scopus, EMBASE, SPORTDiscus, and Cochrane Library databases were searched from inception to 29 June 2024 for systematic reviews reporting total physical activity levels as an exposure and at least one of the above GBD risk factors or BMI as outcomes.

**Results:**

There were 25 systematic reviews that met the inclusion criteria (3 for hypertension, 5 for diabetes, 1 for osteoporosis, and 16 for LDL-cholesterol). Physical activity reduced levels of the risk factors investigated, with dose–response effects observed for blood pressure (6 % for every 600 MET-min/week; 19 % for high versus low activity level) and diabetes (14–28 % if active versus being inactive). Relative to adults not reporting any activity, approximately 600 METs/week reduced levels of LDL-cholesterol by 3.2 % (95 % CI: 1.0 % to 5.4 %) and reduced low bone mineral density by an odds ratio of 0.76 (0.64 to 0.91). No studies of high BMI were identified.

**Conclusion:**

Current risk factor models do not comprehensively assess indirect effects of physical activity through all of the relevant biomedical risk factors. Our study estimated input parameters that can be used to assess these indirect pathways.

## 1 Background

The Global Burden of Disease 2021 (Brauer et al., 2024) updated methods for attributing burden to physical inactivity, and now includes estimates of the attributable burden for all the conditions that are due to high fasting plasma glucose caused by physical inactivity. However, the update only includes burden through the fasting plasma glucose pathway, though there are additional GBD risk factors that physical inactivity influences that in turn effect further disease risk, many of which are not directly linked to physical inactivity.

We have previously outlined an expanded disease and risk factor model for physical inactivity that includes the mediating pathways through these metabolic risk factors (Bourke et al., 2023). Most of these risk factors are associated with diseases that are not usually considered to be caused by physical inactivity. For example, high blood pressure causing chronic kidney disease (Murray et al., 2020). Given that the GBD 2021 new physical inactivity method estimates indirect mediated burden, the full extent to which physical inactivity affects health is potentially underestimated, due to additional conditions that are associated with the other risk factors and are not linked directly to physical inactivity (Bourke et al., 2023). It is important to understand how much additional indirect health impact physical activity might have through these other metabolic risk factors, which first requires quantifying the effect of physical activity on each risk factor – the focus of this current paper.

Evidence for this underestimation can be found by considering the association with all-cause mortality (Atefatfar et al., 2023, Lee et al., 2012, Tarp et al., 2023), which will encompass all pathways and biological mechanisms (i.e: direct and indirect effects). For example, Lee et al. (2012) concluded that all-cause mortality due to inactivity was comparable to that due to smoking or obesity (Lee et al., 2012) – this differs from the rankings of risk factor burden calculated from directly attributed disease deaths, where the deaths from inactivity are 17 % and 10 % of those from smoking and obesity respectively (Murray et al., 2020, Global Burden of Disease Study, xxxx, 2019).

There are many potential sources of error in estimates of causal association. Measurement error in physical inactivity (and the mediating risk factors) will likely underestimate associations, and residual confounding is common. In this current study, we try to be vigilant to these biases, though are largely reliant on the validity of the underlying studies in the systematic reviews.

The objective of this study is to summarize the latest evidence from systematic reviews on the association of physical activity with other risk factors, namely fasting blood plasma glucose (and diabetes, given this disease is also a risk factor for further diseases), blood pressure, cholesterol, and low bone mineral density, and to select the effect size of physical activity to risk factors to use in future modelling of physical activity interventions onto health gains. The scope of risk factors being evaluated in this study is limited to those that are also included in the Global Burden of Disease Study, so that the mediated component of risk factor burden for each risk factor can be estimated. Physical activity may have an effect on a range of biological mechanisms that increase disease risk, including systemic inflammation, insulin resistance, endothelial dysfunction, and triglyceride levels. However, these mechanisms are generally a part of the chain of events involved in the mediation pathways included. While we do include high BMI and obesity in the search strategy, any relationship with physical activity is highly context specific − and in many cases an increase in physical activity is offset with increased energy intake to compensate (Brown et al., 2017, Mytton et al., 2016).

## 2 Methods

This systematic review was not registered in PROSPERO but was performed according to the Preferred Reporting Items for Systematic Reviews and Meta-Analyses (PRISMA) statement (Liberati et al., 2009). Ethics approval was not required for systematic review of publicly available data.

### 2.1 Search strategy

Literature databases (MEDLINE, ProQuest Central, Scopus, EMBASE, SPORTDiscus, and Cochrane Library) were searched from inception (1964, 1938, 2004, 1974, 2007 and 1996 respectively) to 29 June 2024 for English, French and Spanish language systematic literature reviews that reported the relative risk (RR), odds ratio (OR), effect size, or population attributable fraction (PAF) attributed to total physical activity for each risk factor of interest. Search terms included the standard and related/medical terms for (sport OR exercise OR physical activity OR physical exertion) AND (relative risk OR population attributable fraction OR excess risk OR exp risk OR incidence OR effect size OR epidemiology) AND (body mass index OR plasma glucose OR blood pressure OR cholesterol OR bone mineral density) AND (systematic review OR meta-analysis). Search strategy details for each database and MESH terms used are available in Appendix 1.

### 2.2 Eligibility criteria

Published systematic reviews involving adults aged 18 and older with total physical activity as an exposure and any of blood pressure/hypertension, plasma glucose/diabetes, bone mineral density, LDL-cholesterol, or BMI as the outcome. Studied were excluded if they (1) only reported effects of sedentary time, (2) did not measure total physical activity from at least leisure and commuting activity domains (rather than a specific exercise such as cycling), (3) did not report results by physical activity group, (4) did not report a RR, OR, effect size, or enough information to calculate an effect size, (5) only related to clinical subgroups (such as diabetics), (6) were umbrella reviews that only reported reviews already identified, or (7) did not have the full text available in English, French or Spanish. Studies were grouped for analysis by the risk factor under investigation. After conducting the initial review and identifying no eligible studies for cholesterol or bone mineral density, eligibility criteria for these risk factors were modified to include physical activity interventions (mostly systematic reviews of randomized trials) that did not measure total physical activity but did report the activity level of the randomized intervention. Assuming no differential compensatory change in other physical activity between treated and untreated subjects, this allowed us to estimate metabolic equivalent of tasks (METs) and quantify an effect size per unit METs.

### 2.3 Data extraction and analysis

Search results were exported to EndNote and Covidence. Titles and abstracts were independently screened by two reviewers (EB, JR). The full texts of potentially eligible records were assessed against the eligibility criteria by the same reviewers, with additional reviewers (TB, RM) independently assessing those where eligibility was uncertain until group consensus was reached. Reference lists of records selected for full text review were manually searched to identify any additional studies of relevance.

Information from each included systematic review was extracted to identify the: study design (case-control, cohort, etc), number of participants included in meta-analysis, descriptions of variables controlled for to adjust for confounding in underlying studies, physical activity exposure classification and grouping, average length of follow up across underlying studies, and outcome measure classification (e.g. millimetres mercury or a classification of hypertensive for blood pressure). The combined meta-analytic measure (RR, OR, etc) of each systematic review was also extracted.

### 2.4 Meta-analysis

Reference lists were reviewed to identify overlap of included studies, and were selected for inclusion in a meta-analysis based on the publication having less than 10 % overlap with the other remaining publications. A meta-analysis was undertaken using inverse variance to calculate the average change in LDL-cholesterol from the interventions. The meta-analysis included seven publications for cholesterol. Heterogeneity was assessed using the I^2^ statistic.

### 2.5 Assessment of quality of included studies

The quality of systematic reviews was assessed separately for reviews of RCTs (Assessment of Multiple Systematic Review 2 (AMSTAR 2) (Beverley et al., 2017) and cohort studies (JBI Critical Appraisal Checklist for Systematic Reviews and Research Syntheses) (Aromataris et al., 2015). The 16-item AMSTAR 2 checklist assesses areas of weakness across several domains (research question and inclusion criteria, numbers of reviewers involved and databases searched, key words, limits applied to the search, characteristics of included studies, assessments of study quality and publication bias, and conflicts of interest.) rather than provide an overall numerical quality score. The JBI checklist includes 11 items to guide the appraisal of systematic reviews in terms of clarity and appropriateness of the study aims, methods and recommendations.

### 2.6 Selecting the ‘best’ effect size of physical activity to each risk factor

An additional objective of this study was to identify the effect size of physical activity to risk factors to use in future modelling of physical activity interventions onto health gains. While the systematic reviews do not differ much on quality scores, they do differ in terms of utility for modelling. An ideal systematic review would present a meta-analysis of the continuous specification of the risk factor (e.g. millimetres mercury blood pressure, not a dichotomous hypertension variable), with at least three categories of physical activity able to be quantified in terms of a METs dose response; however, no such ideal study existed for any risk factors. Rather, we tabulated systematic reviews by these characteristics (how the outcome is classified (continuous or dichotomous), if the RR is reported by METs/week (as opposed to high vs low), if a dose–response is included, and if a meta-analysis was conducted to obtain the estimate) and selected the systematic reviews with the best match to our criteria.

## 3 Results

### 3.1 Search

**Fig. 1:**
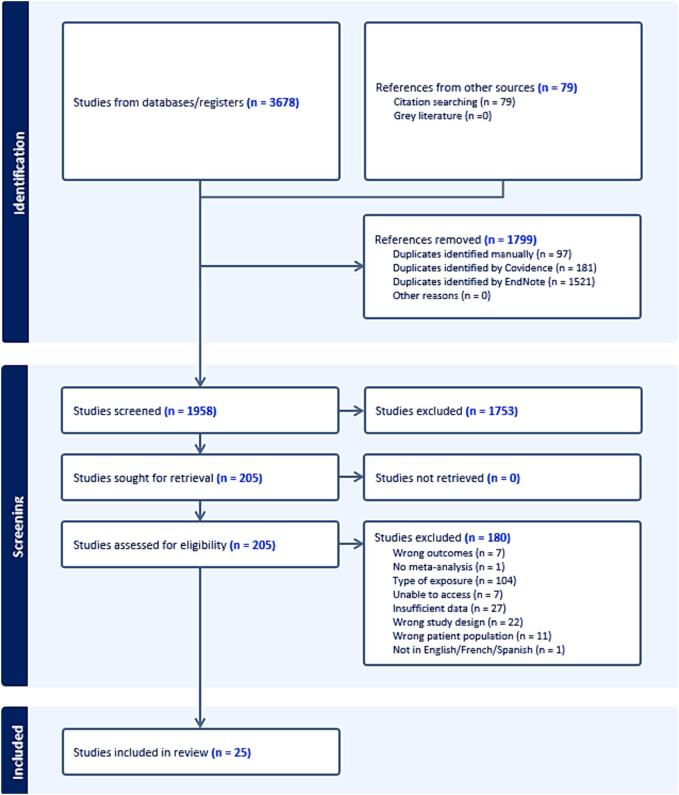
PRISMA diagram for this review of systematic reviews.

The initial search yielded 3,678 published systematic reviews, reduced to 1,958 after removing duplicates (Fig. 1).

The full text review included 205 systematic reviews, and 25 were included. All included reviews for blood pressure and blood glucose reported diagnosis status (hypertension and diabetes) as the outcome rather than risk factor distribution (mean and SD). Of the included reviews, 5 were for diabetes, 3 for hypertension, 1 for bone mineral density, and 16 for cholesterol. No studies for high BMI were identified for physical activity independent of diet, and none were included.

Characteristics of the 25 included reviews are given in Tables 1 (Diabetes and Hypertension) and Table 2 (Osteoporosis and Cholesterol). All reviews were found to be low quality using the AMSTAR 2 domain specific rating system, due to consistently not providing a complete list of excluded studies with justification for exclusion, and not reporting the source of funding for each study included in the review. This system is prone to bias (Li et al., 2022) and so we do not report study ratings in our results. Details of the assessments against AMSTAR 2 criteria and JBI checklist are available in Appendix 2.

**Table 1:** Study characteristics: diabetes and hypertension systematic reviews from prospective cohort studies.

	Publication	Number and type of study	Participants	PA classification	Diagnosis definition	Average follow up	Adjusted for in underlying studies		Relative risk
Diabetes	Aune 2015 (Aune et al., 2015)	Fourteen cohort studies	104,908	Total physical activity − sum ofleisure-time, occupational, and transport activity (high vs low details not stated)	Diabetes based on self-report, physician-diagnosed, record linkage, or medicationuse	13.3 years	Age, sex, BMI, education, smoking status. Inconsistent adjustment for family history, energy intake, alcohol, hypertension, hypercholesterolemia		Low (ref): 1 High versus low total activity: RR 0.65
	Cloostermans 2015 (Cloostermans et al., 2015)	9 prospective cohort studies	117,878	Leisure time and active commuting, classified into low, medium and high (0, 0–150, >150 min/week)	3 studies measured glucose levels, 6 studies self reported	9.1 years	Age, sex, education, smoking, BMI		Low (ref): 1Medium activity: 1.08 (1.04 to 1.13) ,High activity: 1.23 (1.09 to 1.39)
	Kyu 2016 (Kyu et al., 2016)	55 prospective cohort studies	14,051,132 person years	Any activity, converted to METs	Not stated	Not stated	Age, sex, BMI, smoking, family history		0–600 MET-min/week (ref): 1 600–3999: 0.857 (0.816 to 0.902) 4000–7999: 0.748 (0.701 to 0.799)≥8000: 0.722 (0.678 to 0.768)
	Raza 2020 (Raza et al., 2020)	17 prospective cohort studies	1,443,201	Leisure time and active commuting, METs	Not stated	11.2 years	Age, sex, smoking status, alcohol, BP, education level, calorie intake		Low (ref): 1High (not BMI adjusted): 0.78 (0.63 to 0.96)High (BMI adjusted) : 0.82 (0.61 to 1.11)
	Smith 2016 (Smith et al., 2016)	28 prospective cohort studies	1,261,991	Leisure time or total PA converted to METs	Not stated	8.8 years	Age − others inconsistent		Every 10 Met/h-week reduced risk by 0.87 (0.84 to 0.89)
Hypertension	Huai 2013 (Huai et al., 2013)	13 prospective cohort studies	136,846	Leisure time, occupational and communing (separately) − grouped into high/med/low, but cut offs not stated	Measured blood pressure, use of antihypertensive medication, self-report, or from a reimbursement medication registry	9.8 years	age, sex, BMI		Low (ref): 1Moderate: 0.89 (0.85 to 0.94)High: 0.81 (0.76 to 0.85)
	Warburton 2010 (Warburton et al., 2010)	10 prospective cohorts, 1 case-control, 1 cross sectional	113,933	Mix of total physical activity and energy expenditure, tertiles		8.6 years	Not stated		Least active/fit (ref): 1Most active/fit: mean 0.68 (median = 0.70, range 0.37 to 0.90)
	Liu 2017 (Liu et al., 2017)	29 cohort studies	330,222	Leisure time and total PA, converted to METs	Measured blood pressure, use of antihypertensive medication, self-report	Not stated	Age, smoking, alcoholdrinking, education, income, and baseline chronic diseases		RR: 0.94 (0.92 to 0.96) per 10 MET/h-week increase

**Table 2:** Study characteristics: Osteoporosis and cholesterol systematic reviews of randomised controlled trails.

Publication	Number and type of study	Participants	PA classification	Outcome measure	Average follow up	Outcome
Pinheiro, 2020 (Pinheiro et al., 2020)	37 RCTs	1,560	Physical activity vs inactive control intervention − results reported together for any activity type	Bone density at lumbar spine or hip	1 year	Standardised effect size: 0.15 (0.05 to 0.25) (OR 0.76 (0.64 – 0.91))
Cornelissen 2005 (Cornelissen and Fagard, 2005)	30 RCTs	796	Exercise training 1-7xweek, median time 40 min, mostly walking jogging running or cycling	Baseline and change in LDL-cholesterol	16 weeks	Baseline: 3.7 mmol/L±0.54 Change: −0.078 (0.30 to 0.15)
Cornelissen 2011 (Cornelissen et al., 2011)	11 RCTs	NS	Isometric and dynamic resistance training	Baseline and change in LDL-cholesterol	16 weeks	Baseline: 3.2 mmol/L (2.9 to 3.6) Change: −0.082 (−2.3 to 0.084)
Costa 2019 (Costa et al., 2019)	46 RCTs	1,410	Strength training	Baseline and change in LDL-cholesterol	NS	Baseline: NSChange: −0.451 (−0.678 to − 0.224)
He 2023 (He et al., 2023)	24 RCTs	721	Resistance training	Change in LDL-cholesterol	10–26 weeks	Baseline: NS Change: −8.48 mg/dl (−15.05 to −1.91)
Igarashi 2019a (Igarashi et al., 2019)	19 RCTs	597	60–180 min aerobic exercise	Baseline and change in LDL-cholesterol	4–24 weeks	Baseline: 128.4 mg/dL±22.7 Change: −4.3 (−9.4 to 0.8)
Igarashi 2019b (Igarashi and Nogami, 2019)	11 RCTs	353	swimming 15–60 min, 1-4xweek	Baseline and change in LDL-cholesterol	median 12 weeks	Baseline: 144.3 mg/dL±24.2 Change: −10.1 (−18.8 to −1.4)
Kelley 2006 (Kelley and Kelley, 2006)	49 RCTs	2,990	Mostly walking/jogging. Average 3.4xweek, 36.3 min	Baseline and change in LDL-cholesterol	22.3 weeks	Baseline: 141.4 mg/dL±24.4,Change: −3.1 (−1.3 to −4.9)
Kelley 20012a (Kelley and Kelley, 2012)	6 RCTs	788	NS	Baseline and change in non-HDL-cholesterol	NS	Baseline: 160.9 mg/dL±29.3 Change: 3.0 (−7.1 to 13.1)
Kelley 2012b (Kelley et al., 2012)	6 RCTs	192	Average 169 min/week	Baseline and change in LDL-cholesterol	NS	Baseline: 136.0 mg/dL±22.6Change: 2.1 (1.5 to 5.7)
Kelley 2004 (Kelley et al., 2004)	41 RCTs	1,715	Walking, jogging, dance, cycling. 3..7xweek, 36.3 min	Baseline and change in LDL-cholesterol	21.8 weeks	Baseline: 122.1 mg/dL±23.9Change: −4.4 (−3.3 to −5.5)
Kelley 2005 (Kelley et al., 2005)	22 RCTs	948	Walking 4.9xweek, 38.4 min	Baseline and change in non-HDL-cholesterol	22.5 weeks	Baseline Non-HDL: 151.8 mg/dL±21.0 Change: −5.6 (−8.8 to −2.4)
Li 2023 (Li and Zhang, 2023)	7 RCTs	311	NS	Change in LDL-cholesterol	NS	Baseline: NS Change: −0.23 mmol/L (−0.70 to 0.23)
Limbachia 2022 (Thompson and Higgins, 2002)	2	183	NS	Baseline and change in LDL-cholesterol	3 months	Baseline: 2.73 mmol/L Change: −0.02 (−0.10 to 0.05)
Murtagh 2015 (Murtagh et al., 2015)	14 RCTs	664	Walking 20–60 min at 2–7 days per week	Baseline and change in LDL-cholesterol	18.7 weeks	Baseline: 3.72 mmol/L±1.00Change: −0.05 (−0.17 to 0.07)
Xin 2022 (Xin et al., 2022)	18	670	Aerobic, resistance, combined, and water exercise	Baseline and change in LDL-cholesterol	NS	Baseline: 122.36 mg/dL Change: −4.42 (−7.86 to −0.97)
Yun 2023 (Yun et al., 2023)	17	996	Aerobic, resistance, combined, and water exercise	Change in LDL-cholesterol	8–24 weeks	Baseline: NS Change: −0.79 mmol/L (−1.10 to − 0.49)

Meta-analysis was only possible for publications relating to cholesterol (7 of the 16 identified), as overlap of underlying studies was common for diabetes and hypertension reviews (and already conducted within the bone mineral density review).

### 3.2 Physical activity exposure and association with risk factors

**Diabetes or high fasting plasma glucose:** Five reviews included a total of 122 studies (Aune et al., 2015, Cloostermans et al., 2015, Kyu et al., 2016, Raza et al., 2020, Smith et al., 2016) (of which 66 studies were reported in only one review, 38 in two, and 18 in three or more). Physical activity was classified as leisure and commuting time (reported by METs and minutes/week, and grouped into high/medium/low activity; Cloostermans (Cloostermans et al., 2015), Raza (Raza et al., 2020) or leisure, commuting and occupational activities, reported by either METs (Kyu (Kyu et al., 2016), Smith (Smith et al., 2016) or high vs low (Aune (Aune et al., 2015), though details of classification are not stated due to differences in how underlying studies reported results). All reviews reported results from prospective cohort studies.

**Fig. 2:**
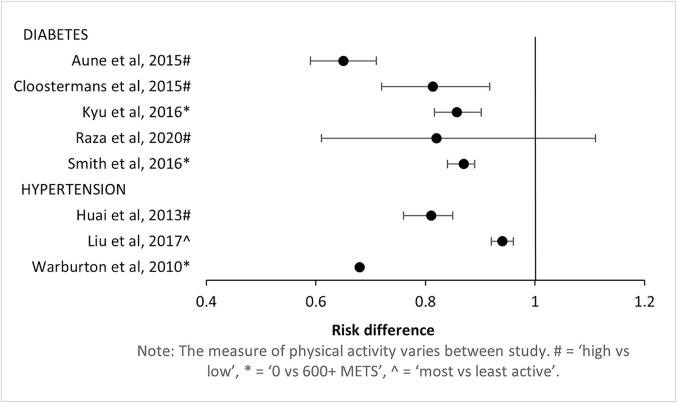
Association between physical activity and risk of diabetes and hypertension.

The outcome was incident diagnosed diabetes, most often identified through self-report, physician diagnosis, or medication received, with an average follow up of 10.5 years (range 8.8 to 13.3 years, Table 1). All reviews found an inverse association between physical activity level and risk of incident diabetes, with relative risks ranging from a mean of 0.65 (Aune (Aune et al., 2015) to 0.87 (Smith (Smith et al., 2016) for high versus low (or the most relevant reported measure) (Fig. 2). Only one review (Raza (Raza et al., 2020) had a 95 % confidence interval range that exceeded 1.0 (to 1.1) for high versus low physical activity. Publication bias was assessed in each of the reviews (except for Cloostermans (Cloostermans et al., 2015) and no evidence of publication bias was identified.

Studies within each systematic review generally adjusted for age, sex, education, smoking, and BMI (Table 1). There was also inconsistent adjustment for other variables such as family history, energy intake, alcohol consumption, hypertension, or hypercholesterolemia. The effect of including/excluding certain potential confounders was tested in one review (Aune (Aune et al., 2015) and little difference in relative risk estimated was identified for most confounders, with the potential exception of hypertension, serum cholesterol, and family history of diabetes.

All of the reviews estimated a dose–response, which was favourably associated in each of these reviews (Aune et al., 2015, Cloostermans et al., 2015, Kyu et al., 2016, Raza et al., 2020, Smith et al., 2016).

**Hypertension:** Three reviews included a total of 34 studies (Huai et al., 2013, Liu et al., 2017, Warburton et al., 2010) (33 prospective cohort, 1 case-control, of which 24 studies were reported in only 1 review, 7 studies in 2 reviews, and 3 in all 3 reviews) and classified physical activity by level of leisure time and total physical activity converted to METs (Liu (Liu et al., 2017), separate categorisation of leisure time, occupational, and commuting activity into high/medium/low (cutoffs not stated; Huai (Huai et al., 2013), or a combination of total physical activity and total energy expenditure by tertiles (Warburton (Warburton et al., 2010). All reviews reported results from prospective cohort studies.

All included hypertension reviews used incident hypertension as the outcome (Fig. 2). This was defined based on measured blood pressure and different cutoff values for systolic/diastolic blood pressure, or through use of antihypertensive medication. Average follow up was 9.2 years (range 8.6 to 9.8 years; Table 1). All reviews showed a protective association between physical activity and risk of hypertension (Fig. 2), with relative risks ranging from a mean of 0.81 (Huai (Huai et al., 2013), high (and moderate) vs low physical activity) to 0.94 (Liu (Liu et al., 2017), 10 MET-hr/week). Warburton (Warburton et al., 2010) reported an average relative risk according to physical activity and fitness; most active/fit vs least active/fit (average RR=0.68, no CI reported), though they did not undertake a meta-analysis. Publication bias was assessed in Huai (Huai et al., 2013) and Liu (Liu et al., 2017), but not (Warburton (Warburton et al., 2010), and no evidence of publication bias was identified.

Adjustment for confounders was inconsistent across individual studies in each systematic review, so outcomes may include some degree of residual confounding. While studies in Huai generally adjusted for age sex and BMI, studies in Liu (Liu et al., 2017) generally adjusted for age, smoking, alcohol, education, income, and baseline chronic disease (Table 1). Liu examined the effect of including/excluding potential confounders, and reported no substantive difference in the effect size for age, smoking, alcohol drinking, baseline chronic disease, education or income.

All 3 reviews found a dose response relationship between physical activity and hypertension risk. Liu (Liu et al., 2017) found a linear dose response, where every 10 MET-hr/week increase in activity reduced hypertension risk by 6 %.

**Osteoporosis (bone mineral density):** One review (Pinheiro et al., 2020) included 52 studies (12 cohort, 40 RCT). Heterogeneity of the cohort studies meant only RCT results were included in a meta-analysis. Physical activity treatments evaluated in RCTs included balance and functional exercises (Beverley et al., 2017), resistance (Aromataris et al., 2015), endurance (Lee et al., 2012), combination exercises (Mytton et al., 2016), and tai chi (Brauer et al., 2024).

The outcome in all RCTs was risk of osteoporosis following physical activity interventions among participants with low physical activity (Table 2). Osteoporosis was identified by bone densitometry at the femoral neck or lumbar spine and was defined as density 2.5 standard deviations or more below optimal levels, with an average follow up period of 1 year. These results showed a standard effect measure of 0.15 (95 % CI: 0.05 to 0.25, equal to an odds ratio of 0.76 (0.64 – 0.91)) from all types of physical activity interventions. This review did not explore the effects of including or excluding confounders. Dose response was assessed but not identified, due to lack of statistical power. Publication bias was assessed and not identified.

**Cholesterol:** Sixteen systematic reviews included 227 RCTs that investigated effects of physical activity on LDL-cholesterol for RCTs (Cornelissen and Fagard, 2005, Cornelissen et al., 2011, Costa et al., 2019, Igarashi et al., 2019, Igarashi and Nogami, 2019, Kelley and Kelley, 2006, Kelley and Kelley, 2012, Kelley et al., 2012, Kelley et al., 2004, Kelley et al., 2005, Murtagh et al., 2015, Limbachia et al., 2022, Xin et al., 2022, He et al., 2023, Li and Zhang, 2023, Yun et al., 2023) (PA intervention vs inactive control). The interventions related to ‘aerobic’ exercise (Tarp et al., 2023), endurance exercise (Bourke et al., 2023), resistance/strength training (Murray et al., 2020), and walking (Bourke et al., 2023). The average level of activity undertaken in the interventions was approximately 600 MET-min/week.

All systematic reviews reported baseline measurements and average change following the intervention. In all but one review, physical activity reduced LDL-cholesterol levels. Publication bias was assessed in all reviews, and was not identified.

**Fig. 3:**
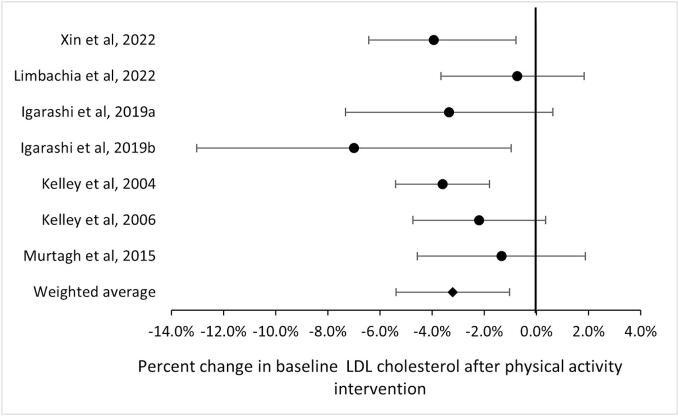
Forest plot of percent change in cholesterol for seven of the systematic reviews and meta-analyses.

Seven reviews (Igarashi et al., 2019, Igarashi and Nogami, 2019, Kelley et al., 2004, Kelley and Kelley, 2006, Murtagh et al., 2015, Limbachia et al., 2022, Xin et al., 2022) selected for inclusion in meta-analysis included 155 RCTs (64 %), and estimates of intervention effects on LDL-cholesterol ranged from −1% to −7%. Heterogeneity between studies was high (I^2^ = 82.6 %). The inverse-variance weighted average was 3.2 % (95 % CI: 1.0 % to 5.4 %). In four of the 7 reviews, the confidence interval crossed the 0 % threshold (null-effect) (Fig. 3). The interventions were associated with relatively small average increases in physical activity levels (moving from a non-active state to a low activity level), and none of the reviews tested for a dose response.

### 3.3 Best effect estimates

Table 3 summarises the reviews utility for incorporation into physical activity models, based on how the outcome is classified (continuous or dichotomous), if the RR is reported by METs/week (as opposed to high vs low), if a dose–response is included, and if a meta-analysis was conducted to obtain the estimate. For blood pressure and blood glucose, at least one review was found for each risk factor that met 3 of these 4 criteria, though all reviews were classified by a dichotomy (diabetes, hypertension) rather than in natural units per level of activity. In the case of high blood glucose, two reviews were identified that met 3 of the criteria, and both report similar results (with the confidence interval from Kyu (Kyu et al., 2016) wholly containing that of Smith (Smith et al., 2016). The review for bone mineral density met only one of the 4 criteria (conducting a meta-analysis). Reviews for cholesterol did not report RR by METs or a dose response, but did report cholesterol as a continuous variable.

**Table 3:** Summary of review attributes for utility for inclusion in physical activity models.

Risk factor	Study	Classification	RR by METs	Dose Response (at least 3 categories)	Meta-analysis	Result
High blood glucose	Kyu et al, 2016 (Kyu et al., 2016)	Dichotomous (diabetes)	Yes	Yes	Yes	**RR: 0.86 (0.82**–0.90)*
	Smith et al, 2016 (Smith et al., 2016)	Dichotomous (diabetes)	Yes	Yes	Yes	RR: 0.87 (0.84–0.89)*
	Aune et al, 2015 (Aune et al., 2015)	Dichotomous (diabetes)	No	Yes	Yes	RR: 0.65 (0.59–0.71)*
	Cloostermans et al, 2015 (Cloostermans et al., 2015)	Dichotomous (diabetes)	No	Yes	Yes	RR: 0.81 (0.72–0.92)*
	Raza et al, 2020 (Raza et al., 2020)	Dichotomous (diabetes)	No	Yes	Yes	RR: 0.82 (0.61–1.11)

High blood pressure	Liu et al, 2017 (Liu et al., 2017)	Dichotomous (hypertension)	Yes	Yes	Yes	**RR: 0.94 (0.92**–0.96)*
	Huai et al, 2013 (Huai et al., 2013)	Dichotomous (hypertension)	No	Yes	Yes	RR: 0.81 (0.76–0.85)
	Warburton et al, 2010 (Warburton et al., 2010)	Dichotomous (hypertension)	No	Yes	Yes	RR: 0.68

Low bone mineral density	Pinheiro et al, 2020 (Pinheiro et al., 2020)	Dichotomous (osteoporosis)	No	No	Yes (RCTs)	**OR: 1.3 (1.1 – 1.6) for active compared to inactive**

Cholesterol	Igarashi et al, 2019 (Igarashi et al., 2019)	Continuous (mmol/L)	No	No	Yes	**LDL cholesterol reduced 3.2 % (1.0 % –5.4 %) following intervention, from inverse variance weighted** meta**-analysis**
	Igarashi et al, 2019 (Igarashi and Nogami, 2019)	Continuous (mmol/L)	No	No	Yes	
	Kelley et al, 2004 (Kelley et al., 2004)	Continuous (mmol/L)	No	No	Yes	
	Kelley et al, 2006 (Kelley and Kelley, 2006)	Continuous (mmol/L)	No	No	Yes	
	Murtagh et al, 2015 (Murtagh et al., 2015)	Continuous (mmol/L)	No	No	Yes	
	Xin et al, 2022 (Xin et al., 2022)	Continuous (mmol/L)	No	No	Yes	
	Limbachia et al, 2022 (Limbachia et al., 2022)	Continuous (mmol/L)	No	No	Yes	

* where a dose–response is reported, the RR is the activity category closest to 600 MET-min/week.

The best evidence for this purpose showed that physical activity (including exercise) reduced the risk of hypertension by 6 % for every 600 MET-min/week (Liu, 2017 (Liu et al., 2017), and diabetes by 14 % for those accumulating at least the recommended volume of MET-minutes per week (Kyu, 2016 (Kyu et al., 2016). Relative to adults not reporting any activity, approximately 600 METs/week reduced the levels of LDL-cholesterol by 3.2 % (95 % CI: 1.0 % to 5.4 %), and reduced the likelihood of having low bone mineral density by an odds ratio of 0.76 (95 % CI: 0.64 – 0.91; Pinheiro, 2020 (Pinheiro et al., 2020).

## 4 Discussion

This review evaluated the evidence from systematic reviews with meta-analyses for the effect of physical activity onto other risk factors, including diabetes (given the absence of reviews for fasting plasma glucose), hypertension (given the absence of reviews for systolic blood pressure), osteoporosis (given the absence of reviews for bone mineral density), and cholesterol. Physical activity (including exercise) reduced the risk of hypertension, diabetes, LDL-cholesterol, and low bone mineral density. Dose-response relationships were consistently observed for hypertension and diabetes, but were not assessed for LDL-cholesterol or bone mineral density.

The risk factors high blood pressure, high fasting plasma glucose, high LDL-cholesterol, and low bone mineral density account for 6.5 %, 6.1 %, 3.4 %, and 1.6 % of total health loss globally respectively (Murray et al., 2020), due to premature mortality and prevalent disease, while the prevalence of physical inactivity is high (28 %)(Global status report on physical activity, 2022). On a population scale, reducing these risk factors by the 3 % to 14 % risk differences from the ‘best estimates’ found in our review could translate to substantial avoided disease burden for those who are inactive.

While no reviews for cholesterol measured total physical activity, many reviews did evaluate intervention effects across randomised trials (Kelley and Kelley, 2006, Cornelissen and Fagard, 2005, Cornelissen et al., 2011, Costa et al., 2019, Igarashi et al., 2019, Igarashi and Nogami, 2019, Kelley and Kelley, 2006, Kelley and Kelley, 2012, Kelley et al., 2012, Kelley et al., 2004, Kelley et al., 2005, Murtagh et al., 2015). As no existing umbrella reviews were identified for this risk factor, we undertook a meta-analysis across five reviews with the least overlap of contributing individual trials (Igarashi et al., 2019, Igarashi and Nogami, 2019, Kelley et al., 2004, Murtagh et al., 2015, Kelley and Kelley, 2006). We estimated a 3.2 % reduction in LDL-cholesterol arising from physical activity interventions, which is a roughly similar magnitude to estimates of risk reduction for hypertension (Liu et al., 2017) and diabetes (Kyu et al., 2016) at a similar level of activity (around 600 MET-min/week). Further studies should be undertaken to evaluate the dose–response of physical activity on cholesterol.

Studies varied in terms of how they measured physical activity, and what types of physical activity were included. Heterogeneity in measurement and classification of physical activity makes comparisons and dose responses difficult to interpret, as outlined by Strain et al 2020 (Strain et al., 2016) and Ainsworth 2015 (Ainsworth et al., 2012). One method to assess the impact of heterogeneity between studies is meta-regression on study-level covariates that might explain heterogeneity, however this is generally not recommended where fewer than 10 studies are included, so was not possible to undertake in our review (Thompson and Higgins, 2002). Physical activity was generally measured using self-reported data (for diabetes and hypertension), rather than accelerometer-based measures, and is therefore likely mismeasured as self-recall data may be less accurate than accelerometer data and overestimate physical activity, or be impacted by varying interpretations of questions (Dyrstad et al., 2014, Sirard et al., 2013, Torres et al., 2021, Schuna et al., 2013). In future, standardised definitions and measurement protocols should be used in studies of physical activity to enable accurate conclusions to be drawn, ideally (for recent exposure) using a combination of accelerometer based measures of activity and self-reported measures where accelerometer data does not capture all activity (such as during swimming and cycling) to reduce measurement biases (Helmerhorst et al., 2012, Troiano et al., 2008). Even so, the association between physical activity and the intermediate risk factor existed across all measures of activity.

Physical activity is a multidimensional exposure, meaning its full contribution to disease risk requires accurate assessment of average physical activity per week by total METs undertaken, and potentially additional consideration of the manner in which activity is performed (such as bouts of high intensity activity (Dempsey et al., 2022), sedentary time (Dempsey et al., 2020, Ekelund et al., 2019), or lifetime exposure to physical activity (Torres et al., 2021, Ekelund et al., 2019, Friedenreich et al., 2009). The field is not yet that advanced, and so we are only considering one dimension of physical activity through average total METs. Within this construct, there is likely substantial measurement error due to the time period of exposure (physical activity generally being measured over the past week, but impacts on health may be cumulative over many years, such as the case for smoking (Jha and Peto, 2014). Not all the systematic reviews included in our study reported the type of physical activity undertaken or the dose, particularly for studies of LDL-cholesterol, and results may be biased if the activities undertaken in the included trials are potentially more or less beneficial for LDL-cholesterol. Data on total physical activity in MET-min/week and a dose response are critical to fully understand the relationship between physical activity and these biomedical risk factors. Data from prospective cohort studies for LDL-cholesterol and bone mineral density would facilitate a more robust analysis of the impact of physical activity, as intervention studies over a short term are unlikely to capture the full scope of effects (Lee et al., 2001). If these measurement errors are non-differential by level of the outcome (in this case, the other risk factors of interest), which seems likely, there will be underestimation of the causal associations reported in our study.

Additionally, residual confounding is almost inevitable, and likely to mean a bias to overestimate the association of physical activity with diseases (i.e. physical activity is less common among smokers meaning incomplete adjustment for smoking will leave positive residual confounding (Zhang et al., 2023). While adjustment for measured confounders was undertaken in all of the studies included in the reviews, not all of the potential confounders were accounted for in all studies. Where confounders adjusted for were reported, age, sex, and BMI were adjusted for in the studies contained within each review in all except one review (Smith 2016 (Smith et al., 2016). Other important confounders (Lee et al., 2012, Bauman et al., 2012) are education, family history, smoking status, and alcohol consumption, and were generally adjusted for. Inconsistencies in confounder adjustment limit the comparability of results, and may lead to bias in the effect sizes estimated. Due to the few reviews identified for each risk factor, meta-regression was unable to be conducted to determine the effect of including or excluding adjustments for certain confounders. The net effect of these conceptual, measurement, and confounding biases (and any potential selection biases) is difficult to determine, but probably leads to underestimation of the total impact of physical activity on other risk factors. Future work should be undertaken to conduct better underlying studies, and use quantitative bias analysis where possible and necessary in underlying studies before they are included in systematic reviews (Lash et al., 2014).

The input studies to the ‘best’ systematic review for hypertension and diabetes are still potentially subject to bias. The reviews pooled estimates over studies that were variably adjusted for confounding. In reviews (Aune (Aune et al., 2015) for diabetes, Liu (Liu et al., 2017) for hypertension) that that did test the effect of adjusting for certain confounders (e.g. sex, age and income), there was mixed evidence of residual confounding: while Liu did not identify any differential effects on their results, Aune tested a larger number of confounders in their heterogeneity analysis than Liu and the findings suggest that inadequate adjustment for family history, hypertension, and serum cholesterol may lead to overestimated effect sizes for diabetes. None of the reviews reported the impact of adjusting for specific confounders within each study (as opposed to across studies that have variably adjusted for them), except for considering the effect of BMI, and did not discuss in detail the likely impact or magnitude of residual confounding. While the input studies to the reviews were moderately well adjusted for confounding, in general, the ‘best’ estimates we present may potentially be overestimated, because of residual confounding to the extent that input studies could not control for all confounders.

This systematic review summarised and estimated the strengths of association for physical inactivity with biomedical risk factors that may be mediators of physical activity effects on disease (and are often potential causes of diseases which are not usually causally associated with physical inactivity). This provides a starting point for further work to understand the magnitude of a fuller set of pathways from physical inactivity to health and disease in risk factor models. Physical activity exposure should be further harmonised to better incorporate estimates into models – as a start, total physical activity status (capturing all activity undertaken in leisure, transport, and occupational domains) should be reported by METs/week, rather than high vs low physical activity. Longer term consideration should be given to any differential effect by the type of activity undertaken (if such an effect is identified). It would be useful if future research on the effect of physical activity onto other risk factors specified risk factors in natural units (e.g. fasting plasma glucose, not diabetes). These estimates would allow estimation of shifts in risk factor distributions for better estimation of total health impacts in intervention models of physical activity to health gain.

### 4.1 Strengths and limitations

The strengths of this study lay in the comparison of many systematic reviews investigating the association of physical activity with a range of risk factors relevant to mediation of health impacts. This provides a broad view of the estimates available to support these associations. In doing so, we assessed the reviews for how useful they may be for further incorporation into comparative risk assessment models, and tried to review the likely impact of bias within these studies.

There are some limitations to our review of reviews. Results for osteoporosis and cholesterol are from intervention studies rather than observational studies, and while this means issues of confounding are removed, it also comes with two key limitations. First, the interventions were specific physical activity interventions, not an overall increase in participants total physical activity. Nevertheless, the MET equivalent of the interventions can be approximated. Second, the trials for cholesterol were for interventions only lasting weeks to months. While LDL-cholesterol responds quickly to statin (Kang et al., 2019) and other drug treatment, the full effect of physical activity on LDL-cholesterol could take longer to be observed.

## 5 Conclusion

Current risk factor modelling studies do not comprehensively assess indirect effects of physical activity through all of the relevant biomedical risk factors. Our study estimated input parameters that can be used to assess these indirect pathways. Inconsistencies in physical activity classification, measurement, reporting, and analysis may also bias effect estimates.

## 6 Consent for publication

Not applicable.

## 7 Ethics approval and consent to participate

Ethics approval is not needed for this publication, as it discusses published works only and does not contain any data or analysis requiring ethics approval.

## Authors contributions

All authors contributed to the conception and design of this publication. Data analysis was undertaken by EB. Primary drafting was undertaken by EB, with RM, JR and TB revising. All authors gave final approval.

## Funding

None.

## CRediT authorship contribution statement

**Emily Bourke:** Writing – original draft, Project administration, Methodology, Formal analysis, Conceptualization. **Jonathan Rawstorn:** Writing – review & editing, Validation, Investigation, Data curation, Conceptualization. **Ralph Maddison:** Writing – review & editing, Supervision, Conceptualization. **Tony Blakely:** Writing – review & editing, Supervision, Methodology, Conceptualization.

## Declaration of competing interest

The authors declare that they have no known competing financial interests or personal relationships that could have appeared to influence the work reported in this paper.

## Data Availability

All data generated or analysed during this study are included in this published article [and its supplementary information files].
