# Supplementary material for: The effects of physical inactivity on other risk factors for chronic disease: A systematic review of reviews
Source: Prev Med Rep. 2024 Aug 22;46:102866. doi: 10.1016/j.pmedr.2024.102866 (PMC11385465; doi:10.1016/j.pmedr.2024.102866)
Supplement: Supplementary Data 1 [file mmc1.docx]

### **Appendix 1: Search Strategy**

**MEDLINE and EMBASE**

("exercise/".tw. OR sport*.tw. OR physical activity.tw. OR physical exertion.tw.) AND ("relative risk*".mp. OR "population attributable fraction".mp. OR "excess risk".mp. OR exp "risk"/ OR "incidence".mp. OR "effect size".mp. OR epidemiology.tw.) AND (body mass index.tw. OR BMI.tw. OR "body mass/".tw. OR plasma glucose.tw. OR blood sugar.tw. OR blood pressure.tw. OR "Blood Pressure/".tw. OR cholesterol.tw. OR "low density lipoprotein cholesterol/".tw. OR bone mineral density.tw.) AND ("systematic review".ti. OR "meta-analysis".ti.)

**SCOPUS**

((TITLE-ABS(exercise)) OR (TITLE-ABS("sport*")) OR (TITLE-ABS("physical activity")) OR (TITLE-ABS("physical exertion"))) AND ((TITLE-ABS-KEY("relative risk")) OR (TITLE-ABS-KEY("relative risk*")) OR (TITLE-ABS-KEY("population attributable fraction")) OR (TITLE-ABS-KEY("excess risk")) OR (TITLE-ABS-KEY("risk")) OR (TITLE-ABS-KEY("incidence")) OR (TITLE-ABS-KEY("effect size")) OR (TITLE-ABS-KEY("epidemiology"))) AND ((TITLE-ABS("body mass index")) OR (TITLE-ABS("BMI")) OR (TITLE-ABS("plasma glucose")) OR (TITLE-ABS("blood sugar")) OR (TITLE-ABS("blood pressure")) OR (TITLE-ABS("cholesterol")) OR (TITLE-ABS("bone mineral density"))) AND ((TITLE("systematic review")) OR (TITLE("meta-analysis”)))

**PROQUEST CENTRAL**

[(pub(exercise) OR ab(exercise) OR pub(physical activity) OR ab(physical activity) OR pub(physical exertion) OR ab(physical exertion) OR pub(sport*) OR ab(sport*)) AND ((relative risk) OR (population attributable fraction) OR (excess risk) OR risk OR incidence OR (effect size) OR epidemiology) AND (pub(systematic review) OR pub(meta-analysis)) AND ((ab(body mass index) OR pub(body mass index) OR ab(BMI) OR pub(BMI) OR ab(plasma glucose) OR pub(plasma glucose) OR ab(blood sugar) OR pub(blood sugar) OR ab(blood pressure) OR pub(blood pressure) OR ab(cholesterol) OR pub(cholesterol)) OR (ab(bone mineral density) OR pub(bone mineral density))](https://www.proquest.com/recentsearches.recentsearchtabview.recentsearchesgridview.scrolledrecentsearchlist.checkdbssearchlink:rerunsearch/BACCA61285794E6APQ/None?site=central&t:ac=RecentSearches) AND (pub(systematic review) OR pub(meta-analysis)))

**SPORTDISCUS**

(TI "exercise" OR AB "exercise" OR TI "physical activity" OR AB "physical activity" OR TI "physical exertion" OR AB "physical exertion" OR TI "sport*" OR AB "sport*") AND ("relative risk" OR "population attributable fraction" OR "excess risk" OR "risk" OR "incidence" OR "effect size" OR "epidemiology") AND (AB "body mass index" OR AB "BMI" OR AB "plasma glucose" OR AB "blood sugar" OR AB "blood pressure" OR AB "cholesterol" OR AB "bone mineral density" OR TI "body mass index" OR TI "BMI" OR TI "plasma glucose" OR TI "blood sugar" OR TI "blood pressure" OR TI "cholesterol" OR TI "bone mineral density") AND (TI "systematic review" OR TI "meta-analysis")­­

**COCHRANE LIBRARY**

((exercise):ti OR (sport*):ti OR (physical activity):ti OR (physical exertion):ti OR (exercise):ab OR (sport*):ab OR (physical activity):ab OR (physical exertion):ab) AND ((relative risk):ti,ab,kw OR (population attributable fraction):ti,ab,kw OR (excess risk):ti,ab,kw OR (risk):ti,ab,kw OR (incidence):ti,ab,kw OR (effect size):ti,ab,kw OR (epidemiology):ti,ab,kw) AND ((blood pressure):ab OR (cholesterol):ab OR (low density lipoprotein cholesterol):ab OR (bone mineral density):ab OR (blood pressure):ti OR (cholesterol):ti OR (low density lipoprotein cholesterol):ti OR (bone mineral density):ti OR (body mass):ti OR (body mass index):ti OR (BMI):ti OR (plasma glucose):ti OR (blood sugar):ti OR (body mass):ab OR (body mass index):ab OR (BMI):ab OR (plasma glucose):ab OR (blood sugar):ab) AND ((systematic review):ti OR (meta-analysis):ti)

**Appendix 2: Critical appraisal checklists**

**Table A2.1: Critical appraisal of diabetes and hypertension studies using the Joanna Briggs Institute checklist for systematic reviews**

|  | Diabetes | | | | | Hypertension | | |
| --- | --- | --- | --- | --- | --- | --- | --- | --- |
| JBI Checklist | **Aune 2015** | **Cloostermans 2015** | **Kyu 2016** | **Raza 2020** | **Smith 2016** | **Huai 2013** | **Warburton 2010** | **Liu 2017** |
| 1. Is the review question clearly and explicitly stated? | Yes | Yes | Yes | Yes | Yes | Yes | Yes | Yes |
| 2. Were the inclusion criteria appropriate for the review question? | Yes | Yes | Yes | Yes | Yes | Yes | Yes | Yes |
| 3. Was the search strategy appropriate? | Yes | Yes | Yes | Yes | Yes | Yes | Yes | Yes |
| 4. Were the sources and resources used to search for studies adequate? | Yes | Yes | Yes | Yes | Yes | Yes | Yes | Yes |
| 5. Were the criteria for appraising studies appropriate? | Yes | Yes | Yes | Yes | Yes | Yes | Yes | Yes |
| 6. Was critical appraisal conducted by two or more reviewers independently? | Unclear | Unclear | Unclear | Unclear | Yes | Yes | Unclear | Yes |
| 7. Were there methods to minimize errors in data extraction? | Unclear | Yes | Yes | Unclear | Yes | Yes | Yes | Yes |
| 8. Were the methods used to combine studies appropriate? | Yes | Yes | Yes | Yes | Yes | Yes | Unclear | Yes |
| 9. Was the likelihood of publication bias assessed? | Yes | No | Yes | Yes | Yes | Yes | No | Yes |
| 10. Were recommendations for policy and/or practice supported by the reported data? | Yes | Yes | Yes | Yes | Yes | Yes | Yes | Yes |
| 11. Were the specific directives for new research appropriate? | Yes | Yes | Yes | No (no specific directives made) | Yes | Yes | Yes | Yes |

**Table A2.2: AMSTAR 2 quality scores for cholesterol and bone mineral density systematic reviews of RCTs (continues…)**

|  | Cholesterol | | | | | | | |
| --- | --- | --- | --- | --- | --- | --- | --- | --- |
| Amstar Questions | **Cornelissen 2005** | **Cornelissen 2011** | **Costa 2019** | **He 2023** | **Igarashi 2019a** | **Igarashi 2019b** | **Kelley 2006** | **Kelley 2012a** |
| 1. Did the research questions and inclusion criteria for the review include the components of PICO? | Yes | Yes | Yes | Yes | Yes | Yes | Yes | Yes |
| 2. Did the report of the review contain an explicit statement that the review methods were established prior to the conduct of the review and did the report justify any significant deviations from the protocol? | Partial yes | Partial yes | Yes | Yes | Yes | Yes | Partial yes | Partial yes |
| 3. Did the review authors explain their selection of the study designs for inclusion in the review? | Yes | Yes | Yes | Partial yes | Yes | Yes | Yes | Yes |
| 4. Did the review authors use a comprehensive literature search strategy? | Partial yes | Yes | Partial yes | Yes | Yes | Yes | Yes | Yes |
| 5. Did the review authors perform study selection in duplicate? | No | Yes | Yes | Yes | Yes | Yes | Yes | Yes |
| 6. Did the review authors perform data extraction in duplicate? | Yes | Yes | Yes | Yes | Yes | Yes | Yes | Yes |
| 7. Did the review authors provide a list of excluded studies and justify the exclusions? | No | No | No | No | No | No | No | No |
| 8. Did the review authors describe the included studies in adequate detail? | Partial yes | Yes | Yes | Yes | Yes | Yes | Yes | Yes |
| 9. Did the review authors use a satisfactory technique for assessing the risk of bias (RoB) in individual studies that were included in the review? | No | Partial yes | Yes | Yes | Yes | Yes | Partial yes | Yes |
| 10. Did the review authors report on the sources of funding for the studies included in the review? | No | No | No | No | No | No | No | No |
| 11. If meta-analysis was performed did the review authors use appropriate methods for statistical combination of results? | Yes | Yes | Yes | Yes | Yes | Yes | Yes | Yes |
| 12. If meta-analysis was performed, did the review authors assess the potential impact of RoB in individual studies on the results of the meta-analysis or other evidence synthesis? | No | Yes | Yes | No | Yes | Yes | No | No |
| 13. Did the review authors account for RoB in individual studies when interpreting/ discussing the results of the review? | No | No | Yes | No | Yes | Yes | No | No |
| 14. Did the review authors provide a satisfactory explanation for, and discussion of, any heterogeneity observed in the results of the review? | No | Yes | Yes | Yes | Yes | Yes | Yes | Yes |
| 15. If they performed quantitative synthesis did the review authors carry out an adequate investigation of publication bias (small study bias) and discuss its likely impact on the results of the review? | Yes | Yes | yes | Yes | Yes | Yes | Yes | Yes |
| 16. Did the review authors report any potential sources of conflict of interest, including any funding they received for conducting the review? | Yes | Yes | Yes | Yes | Yes | Yes | Yes | Yes |
| Quality rating | **Critically low** | **Critically low** | **Low** | **Critically low** | **Low** | **Low** | **Critically low** | **Critically low** |

**Table A2.2 (continued): AMSTAR 2 quality scores for cholesterol and bone mineral density systematic reviews of RCTs**

|  | Cholesterol | | | | | | | | Bone mineral density |
| --- | --- | --- | --- | --- | --- | --- | --- | --- | --- |
| Amstar Questions | **Kelley 2012b** | **Kelley 2004** | **Kelley 2005** | **Li 2023** | **Limbachia 2013** | **Murtagh 2015** | **Xin 2022** | **Yun 2023** | **Pinheiro 2020** |
| 1. Did the research questions and inclusion criteria for the review include the components of PICO? | Yes | Yes | Yes | Yes | Yes | Yes | Yes | Yes | Yes |
| 2. Did the report of the review contain an explicit statement that the review methods were established prior to the conduct of the review and did the report justify any significant deviations from the protocol? | Partial yes | Partial yes | Partial yes | Yes | Yes | Partial yes | Yes | Yes | Partial yes |
| 3. Did the review authors explain their selection of the study designs for inclusion in the review? | Yes | Yes | Yes | Yes | Yes | Yes | Yes | Yes | Yes |
| 4. Did the review authors use a comprehensive literature search strategy? | Yes | Yes | Yes | Partial yes | Yes | Partial yes | Yes | Yes | Partial yes |
| 5. Did the review authors perform study selection in duplicate? | Yes | Yes | Yes | Yes | Yes | Yes | Yes | Yes | Yes |
| 6. Did the review authors perform data extraction in duplicate? | Yes | Yes | Yes | Yes | Yes | Yes | Yes | Yes | Yes |
| 7. Did the review authors provide a list of excluded studies and justify the exclusions? | No | No | No | No | Yes | No | No | No | No |
| 8. Did the review authors describe the included studies in adequate detail? | Yes | Yes | Partial yes | Partial yes | Yes | Yes | Yes | Partial yes | Yes |
| 9. Did the review authors use a satisfactory technique for assessing the risk of bias (RoB) in individual studies that were included in the review? | Yes | Partial yes | Partial yes | Yes | Yes | Yes | Yes | Yes | Yes |
| 10. Did the review authors report on the sources of funding for the studies included in the review? | No | No | No | No | No | No | No | No | No |
| 11. If meta-analysis was performed did the review authors use appropriate methods for statistical combination of results? | Yes | Yes | Yes | Yes | Yes | Yes | Yes | Yes | Yes |
| 12. If meta-analysis was performed, did the review authors assess the potential impact of RoB in individual studies on the results of the meta-analysis or other evidence synthesis? | No | Yes | No | Yes | Yes | Yes | No | Yes | Yes |
| 13. Did the review authors account for RoB in individual studies when interpreting/ discussing the results of the review? | Yes | Yes | No | Yes | No | Yes | No | Yes | Yes |
| 14. Did the review authors provide a satisfactory explanation for, and discussion of, any heterogeneity observed in the results of the review? | Yes | Yes | No | Yes | Yes | Yes | Yes | Yes | Yes |
| 15. If they performed quantitative synthesis did the review authors carry out an adequate investigation of publication bias (small study bias) and discuss its likely impact on the results of the review? | Yes | Yes | Yes | Yes | Yes | Yes | Yes | Yes | Yes |
| 16. Did the review authors report any potential sources of conflict of interest, including any funding they received for conducting the review? | Yes | Yes | Yes | Yes | Yes | Yes | Yes | Yes | Yes |
| Quality rating | **Low** | **Low** | **Critically low** | **Low** | **Low** | **Low** | **Low** | **Critically low** | **Low** |
